# Exploring the Social, Emotional, and Physical Consequences of Hidradenitis Suppurativa in Pediatric Patients: A Scoping Review

**DOI:** 10.3390/pediatric18040103

**Published:** 2026-08-04

**Authors:** Kathryn Lotharius, Kendell Lewis, Clarissa Portocarrero, Megha Srivastav, Silvia Zervos, Rebecca Urbonas, Michelle Knecht, Lea Sacca

**Affiliations:** Department of Population Health, Charles E. Schmidt College of Medicine, Florida Atlantic University, Boca Raton, FL 33431, USAszervos2024@health.fau.edu (S.Z.); kebam@health.fau.edu (M.K.)

**Keywords:** hidradenitis suppurativa (HS), social impact, emotional consequences, children, mental health

## Abstract

There remains a widespread lack of knowledge regarding hidradenitis suppurativa (HS) among physicians in the United States, impeding timely diagnosis and implementation of comprehensive treatment interventions. Despite the presence of supporting communities for affected adolescents and their caretakers, the overall awareness of HS remains low, and a greater consensus on the treatment of HS is needed. Current research highlights the lack of standardized pediatric guidelines for treatment of HS largely due to the varied nature of the disease and limited efficacy of current therapies. Our study aims to explore the relationship between the chronic skin condition HS and social–emotional concerns, mental health, and physical health issues in US children and adolescents. Using the Arksey and O’Malley framework and PRISMA-ScR reporting, we searched PubMed/MEDLINE, Scopus, Web of Science, Cochrane Library, and Embase for U.S. studies (2015–2025) on pediatric HS (<18 years) and social–emotional, mental health, or quality-of-life outcomes. Recommendations were synthesized, and study quality was appraised with CASP checklist methods and rigor. Ten studies (2020–2025) met inclusion criteria. Pediatric HS was associated with depression, anxiety, social withdrawal, shame, low self-esteem, and reduced quality of life. Physical comorbidities increased psychosocial burden. Socioeconomic and racial disparities worsened outcomes and access to care. Studies emphasized early diagnosis, routine screening, multidisciplinary management, and disparity-focused interventions. Findings may inform clinical practice and guide research initiatives aimed at improving outcomes for children and adolescents with HS.

## 1. Introduction

Hidradenitis suppurativa (HS) is a chronic, relapsing inflammatory disease of the hair follicles primarily affecting apocrine glands in intertriginous areas such as the axillae, inguinal, perianal, mammary, and inframammary regions [1]. Occlusion of hair follicles leads to the development of tender subcutaneous nodules that frequently rupture and progress through cycles of inflammation, healing, and scarring [1,2]. HS is increasingly recognized in pediatric population groups, particularly among female patients, with nearly one-third of cases occurring in childhood [3]. Due to the cyclical nature of flares, diagnosis of HS is often delayed, making it difficult to identify the disease in childhood. In the United States, the prevalence of pediatric HS is estimated at 0.03%, with variation by age [3]. The highest prevalence has been reported among females aged 15 to 17 years who identify as African American (0.53%) or biracial (0.25%) [3].

Contributing factors associated with the development and clinical burden of HS in pediatric populations are multifactorial and include genetic predisposition, comorbid conditions such as obesity and endocrine disorders, and delays in diagnosis and treatment initiation. Evidence suggests a strong hereditary component of HS, with 34% of first-degree relatives of affected individuals also diagnosed with HS [1]. Furthermore, genetic studies have identified autosomal dominant inheritance patterns in certain types of HS [1]. Metabolic comorbidities are highly prevalent among pediatric HS, with affected patients being 2.48 times more likely to be obese when compared to their peers, reflecting a combination of chronic inflammation and mechanical effects of obesity on follicular occlusion [4]. Although HS prevalence remains lower in pediatric patients compared to adults, diagnostic delays in children and adolescents may contribute to prolonged disease progression, increased severity at presentation, and greater psychosocial burden. Early lesions in pediatric patients are frequently misdiagnosed as recurrent infections or acne, resulting in a nearly 2-year gap between symptom onset and diagnosis of HS [3]. Limited access to specialized dermatologic care adds to this issue, particularly in low-socioeconomic status (SES) neighborhoods. Studies have shown an association between living in a low-SES neighborhood and new HS diagnosis, particularly among Black and Latino populations who disproportionately experience barriers to healthcare [5]. These social determinants of health, combined with genetic and clinical characteristics of HS, emphasize the need for early recognition and improved access to dermatologic care in pediatric HS.

Apart from its physical manifestations, HS imposes a psychological and social burden on pediatric and adolescent populations [6]. Children with highly painful, draining lesions or significant scarring often experience stigma, bullying, and social alienation [6]. Quality-of-life studies report that pediatric patients with HS demonstrate greater emotional impairment and reduced participation in school compared to healthy peers [6]. The relapsing nature of HS, coupled with frequent misdiagnosis and mismanagement of the condition, exacerbates feelings of hopelessness and disrupts psychosocial development of children [6]. Consequently, pediatric HS requires treatment from a multidisciplinary stance, integrating dermatologic care with psychological and social support.

There remains a widespread lack of knowledge regarding HS among physicians in the United States, impeding timely diagnosis and implementation of comprehensive treatment interventions [7]. Despite the presence of supporting communities for affected adolescents and their caretakers, the overall awareness of HS remains low, and a greater consensus on treatment of HS is needed [8]. Current research highlights the lack of standardized pediatric guidelines for treatment of HS largely due to the varied nature of the disease and limited efficacy of current therapies [7]. Moreover, although the clinical and psychosocial consequences of HS are not unique to the United States, the context in which these consequences are recognized and managed may differ across healthcare systems. The U.S. setting is characterized by substantial variation in insurance coverage, access to pediatric dermatology and mental health services, neighborhood-level socioeconomic conditions, and racial and ethnic disparities in healthcare access and utilization [5]. These structural factors may influence diagnostic delay, continuity of care, comorbidity recognition, and the psychosocial burden experienced by pediatric patients with HS. Accordingly, this review focused on U.S.-based evidence to examine pediatric HS consequences within a shared healthcare and social context rather than to suggest that the underlying consequences of HS are biologically unique to U.S. patients. Our study aims to explore the relationship between the chronic skin condition HS and social–emotional concerns, mental health, and physical health issues in US children and adolescents. Our findings will highlight the gaps in educational, policy, and resource-oriented interventions to prevent a further increase in trends of dermatologic pathologies similar to HS, along with the identification of areas of concern in children and adolescents’ emotional and mental health stability leading to new emerging comorbidities.

## 2. Methods

This scoping review was conducted using the Arksey and O’Malley framework, which is composed of a five-step process [9]: (1) identify research questions; (2) search for relevant studies; (3) select studies relevant to the research questions; (4) chart the data; and (5) collate, summarize, and report results. The Joanna Briggs Institute (JBI) recommendations guided the data extraction, evaluation, and presentation of results [10]. The Preferred Reporting Items for Systematic Reviews and Meta-Analyses Extension for Scoping Reviews (PRISMA-ScR) checklist was used as a reference checklist for this review [11]. The PRISMA 2020 checklist is provided as Supplementary Materials. The review protocol was registered on Open Science Framework (OSF) at: https://osf.io/s7nfj [created on 22 December 2025].

**Step 1: Identify Research Questions**

Three guiding research questions were used for this scoping review: (1) What is the relationship between the chronic skin condition HS and social–emotional concerns? (2) How are children and adolescents impacted by HS? (3) What can be done to support the mental health and social–emotional development of children and adolescents with HS?

Outcome Framework

The primary outcome domain of this scoping review was the social–emotional and mental health burden associated with pediatric HS. Outcomes of interest included depression, anxiety, suicidal ideation, stigma, shame or embarrassment, social withdrawal, impaired self-esteem, disruption of school or social participation, and reduced quality of life. Secondary outcome domains included physical symptoms and comorbidities, such as chronic pain, obesity, metabolic and endocrine disorders, and other inflammatory conditions, when reported as contributing to or contextualizing the overall psychosocial burden of HS. Given the exploratory purpose of a scoping review, these domains were used to organize and map the available evidence rather than to define a single effectiveness endpoint.

**Step 2: Search for Relevant Studies**

Keywords and search terms were developed in collaboration with a research librarian (MK) experienced in scoping review methodologies. The search terms included: *hidradenitis suppurativa*, *social-emotional*, *mental health*, *quality of life*, *pediatric dermatology*, *and chronic skin disorder*. Searches were performed using five databases (PubMed including Medline, Embase, Cochrane Library, Web of Science, and Scopus) and a review of the literature took place from June 2025 to August 2025. The screening process was completed using Covidence software [12]. Screening of the titles and abstracts of the articles was initially carried out by one pair of co-authors (KL and RU) and conflicts were resolved by the senior author (LS). Full-text screening was then carried out by the primary author (KL) and senior author (LS).

***Inclusion Criteria.*** The articles included in this review were peer-reviewed descriptive, observational, review, and experimental studies published between 2015 and 2025 that examined associations between HS and social–emotional well-being in pediatric populations in the United States. The review was restricted to U.S.-based studies to maintain a defined healthcare and social context for examining access to specialty care, socioeconomic inequities, and racial and ethnic disparities relevant to pediatric HS. Studies were eligible if they specifically examined pediatric or adolescent HS populations. Studies using broader age ranges were retained only when the study was explicitly pediatric-focused or when pediatric findings were reported within the study framework. Eligible evidence sources included primary empirical studies examining social–emotional, mental health, quality-of-life, or related physical consequences of pediatric HS. Given the limited pediatric-specific literature and the scoping objective of identifying recommendations for clinical care and future research, selected narrative reviews and expert commentary were additionally retained as contextual evidence sources when they provided substantive pediatric-specific discussion.

**Exclusion Criteria.** Excluded studies were those not written in English, published abstracts, and articles that did not address HS or the social–emotional consequences, mental health, or quality of life. Studies conducted outside the U.S. were also excluded.

**Step 3: Selecting Studies Relevant to the Research Questions**

Data were extracted and summarized by co-authors (KL and RU) and any discrepancies were resolved by the senior author (LS) through discussion to reach a consensus.

**Steps 4 and 5: Data Charting, and Collation, Summarization, and Reporting of Results**

Table 1 organized the study characteristics by the primary author and year, study design, sample size, study population, U.S. location, age range, study purpose, social and emotional/physical concerns, and major health outcomes related to HS. Table 2 summarized the associations between HS and social–emotional concerns, including the statistical analyses applied, the significance of association, and the database used. Table 3 presented recommendations from the included studies to inform future research, public health initiatives, and regulatory guidance surrounding pediatric HS. For Table 3, the research team applied the three phases of qualitative content analysis described by Elo and Kyngäs [13,14] (2008): (i) preparation, (ii) organizing, and (iii) reporting. In the preparation phase, a theme was identified as the unit of analysis. The organizing phase involved open coding of qualitative data extracted from the included studies, the creation of general categories, and abstraction into higher-order classifications. The final step was reporting the synthesized recommendations in Table 3 [13,14].

## 3. Results

**Figure 1:**
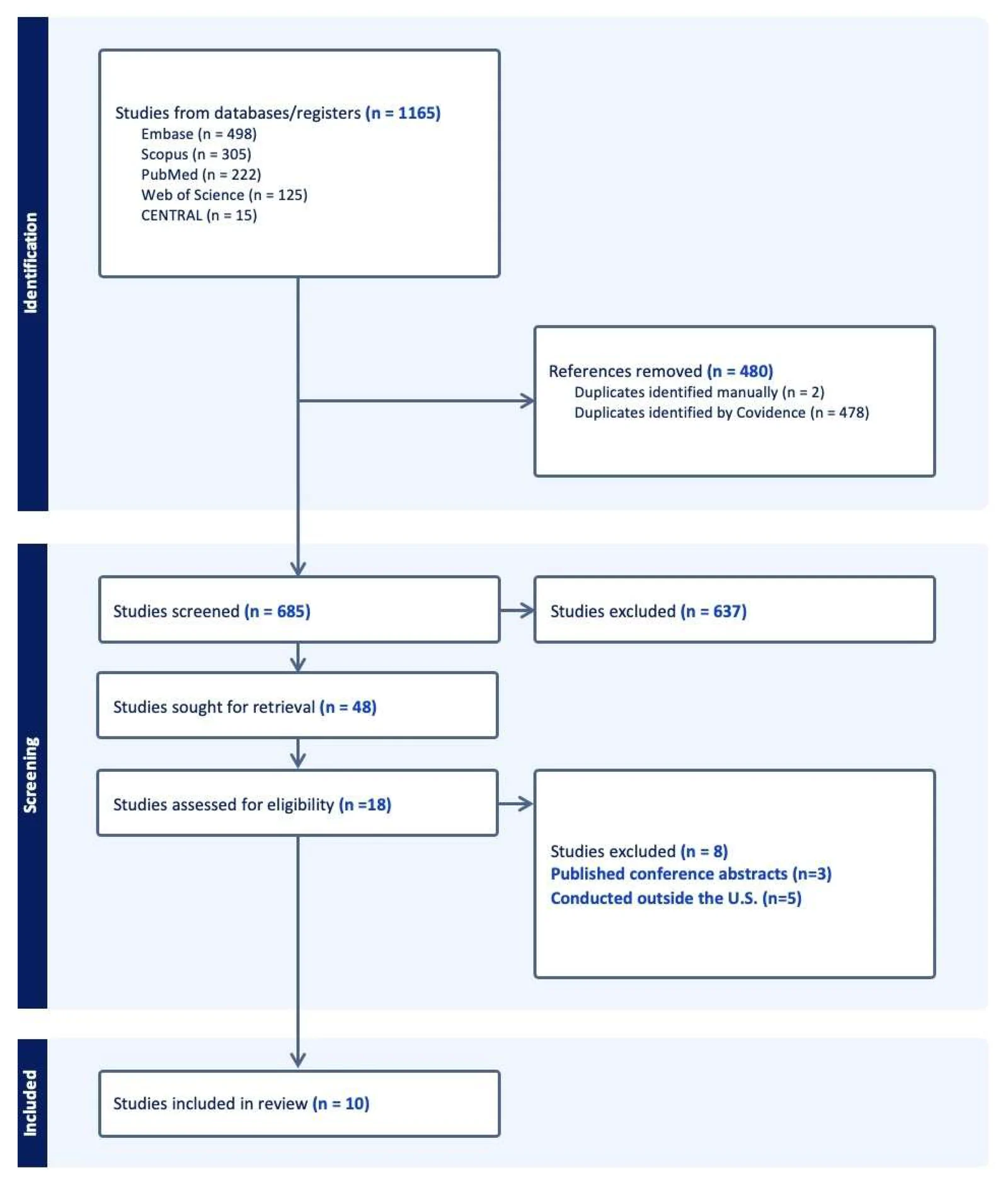
PRISMA Flow Chart.

A total of 10 eligible studies, published between 2020 and 2025, were included. Study designs included cohort studies (*n* = 5), narrative reviews (*n* = 3), one cross-sectional study (*n* = 1), and one editorial (*n* = 1) (Figure 1). To avoid conflating levels of evidence, quantitative associations and empirical outcome estimates were synthesized from primary studies, while non-primary sources were used to contextualize clinical recommendations, research gaps, and broader implications for pediatric HS care. The sample size across studies ranged from *n* = 9 to *n* = 5907 participants. Study populations included pediatric patients with hidradenitis suppurativa (HS) within the US, with state locations encompassing California (*n* = 3), New York (*n* = 1), Washington DC (*n* = 1), Louisiana (*n* = 1), Pennsylvania (*n* = 1), or a combination of states through nationwide datasets (*n* = 3).

### 3.1. Primary Outcome Domain: Social, Emotional, and Mental Health Consequences of HS

Social concerns were identified in nine of the included studies [3,15,17,18,19,20,21,22,23]. The most frequent social concern identified was peer-related stigma, shame, and embarrassment (*n* = 5), whereby adolescents reported social withdrawal and judgment from peers due to visible lesions, odor, or non-adherence to social norms like shaving [3,15,17,18,19]. Limited participation in sports, school, and career opportunities was also reported (*n* = 3), reflecting both physical manifestations and self-consciousness regarding disease visibility [3,19,20]. Furthermore, increased rates of alcohol, smoking, and substance use were identified as a social consequence (*n* = 2), while socioeconomic and racial differences were highlighted as major factors affecting access to dermatologic care and worsening social outcomes, particularly among low-income populations [17,20,21,22]. Finally, disease burden was found to impact family members in one study, thereby affecting family relationships [17]. Emotional concerns were mentioned across all included studies [3,15,17,18,19,20,21,22,23]. The most frequently reported finding was depression (*n* =9), followed by anxiety (*n* =5) and suicidal ideation (*n* = 2) [3,15,16,17,18,19,20,21,22,23]. Overall, poor mental health was disclosed as an emotional burden, with feelings of hopelessness, frustration, and poor self-esteem being reported consistently (*n* = 4) [18,19,20,21]. Substance use disorder (*n* = 1), emotional distress among family members caring for children with HS (*n* = 1), and impaired emotional development (*n* = 1) were also reported as frequently experienced emotional challenges [3,15,20]. As for psychosocial outcomes, several studies reported health comorbidities. For example, Cohn et al. identified an increased risk of anxiety disorders and functional impairment related to psychosocial distress that may persist into adulthood [15]. Furthermore, Wright et al., in both their 2020 and 2021 studies, reported a high prevalence of depression and anxiety, with three new cases of depression diagnosed each year for every 100 patients with HS without preexisting depression [22,23]. Consistent with these findings, Cotton et al. and Power et al. described an overall reduced quality of life and mental health among affected adolescents [3,19].

### 3.2. Secondary Outcome Domain: Physical Symptoms and Comorbidities

Physical concerns were reported in seven of the 10 included studies [3,15,16,20,21,22,23]. In particular, metabolic and endocrine comorbidities, including obesity and polycystic ovary syndrome (PCOS), were highlighted in four studies, while pulmonary diseases such as asthma were reported in two studies [20,21,22,23]. The second most frequently described finding was pain (*n* = 4), often associated with abscess formation or treatment, and potential disfigurement [3,15,16,21]. As for health outcomes, Cotton et al. and Ghanshani et al. identified associations between HS and obesity, PCOS, diabetes, inflammatory bowel disease (IBD), hypertension, and dyslipidemia [3,16]. Ghanshani et al. further noted increased rates of inflammatory conditions, including spondylarthritis, while Cotton et al. noted additional comorbidities such as psychiatric disorders, Down syndrome, and other skin conditions in pediatric populations with HS [3,16]. Seivright et al. further elaborated on prevalent metabolic and dermatologic comorbidities, including acne vulgaris, acanthosis nigricans, and atopic dermatitis [20]. Similarly, Kirby et al. and Lee et al. found that pediatric patients commonly presented with obesity and related comorbidities, with Kirby et al. additionally describing hormonal imbalances leading to PCOS and precocious puberty [17,18]. Lastly, the remaining physical outcomes described included chronic pain and decreased range of motion [18,19].

### 3.3. Association Between HS and Social–Emotional Concerns

Of the 60.0% (*n* = 6) studies that did include statistical analysis, methods used to analyze the association between HS and social–emotional concerns ranged from Cox proportional hazards regression analysis (*n* = 2) to logistic regression analysis (*n* = 2), chi-squared tests and *t*-tests (*n* = 4), complete case analysis (*n* = 1), and propensity score matching (*n* = 1) [15,19,20,21,22,23]. Additionally, five of these studies reported statistically significant associations between HS and at least one social–emotional concern (Table 2) [15,19,20,21,22,23]. Specifically, 60.0% (*n* = 3/5) of studies showed associations with history of depression or adult onset depression and obesity, 40.0% (*n* = 2/5) showed associations with tobacco or substance use disorders, asthma, and anxiety, and 20.0% (*n* = 1/5) showed associations with female sex, Black race, acne vulgaris, acanthosis nigricans, seborrheic dermatitis, pilonidal cysts, psoriasis, down syndrome, irregular periods, PCOS, hirsutism, precocious puberty, elevated serum testosterone, pre-diabetes, diabetes, suicidal ideations, and cutaneous absesses [15,20,21,22,23]. Of the non-significant variables related to HS, 50.0% (*n* = 3/6) displayed associations with BMI, 33.3% (*n* = 2/6) displayed associations with age, insurance type, frequency of ED visits related to HS, history of depression, and hypertension, and 16.7% (*n* = 1/6) displayed associations with gender, age at diagnosis, duration of disease prior to surgical encounter, incision and drainage procedures, atopic dermatitis, hypercholesterolemia, ADHD, autism, bipolar disorder, anemia, sepsis, PCOS, pilonidal cysts, and substance use disorder (Table 2) [15,19,20,21,22,23].

Various databases or hospital electronic medical records were used to collect data on HS. These databases include the IBM Explorys Life Sciences Database (*n* = 3), personal hospital electronic medical records (Children’s Hospital of New Orleans and the University of California Los Angeles) (*n* = 2), and the 2016 Kids’ Inpatient Database (KID) (*n* = 1) (Table 2) [15,19,20,21,22,23].

**Recommendations and Future Directions**

Thematic content analysis of studies underscored the importance of contributing sufficient research and resources towards advancing HS pediatrics-based knowledge, initiating quality-of-life and psychosocial screenings, advocating for low-income and underserved population groups affected with HS, and raising awareness of this chronic pediatric dermatologic disease.

### 3.4. Pediatric-Focused Research

Many studies highlighted the lack of modern research on HS in pediatric populations [3,17,18,20]. The diagnosis of HS commonly occurs in early teenage years through young adulthood; however, the studies in this scoping review consistently highlight the lack of research specific to pediatric populations [3,17,18,20]. Pediatric-focused research may help mitigate the challenges imposed by a diagnosis of HS by equipping families and providers with critical information to address the disease at its early stages [3,17]. Furthermore, incorporating prospective, long-term follow-up interactions would help further the discussion on implementation efforts to properly manage HS in pediatric patients in clinical and home settings, including beneficial modalities to explore for sustained impact (Table 3) [3,17].

### 3.5. Initiating Early Screening Practices

A significant barrier to receiving adequate HS-tailored care is overlooking the psychosocial comorbidities that may accompany this diagnosis [15,16,17,20]. Adolescents diagnosed with HS should receive screening for anxiety disorders, suicidal ideation, and depression to aid in the early implementation of evidence-based interventions to help detect and mitigate the impact of these mental health comorbidities [3,15,16,17,18,19,20,21,22,23]. In addition, increased screening for metabolic and endocrine comorbidities should be considered given the documented burden of obesity, dyslipidemia, glycemic abnormalities, PCOS, and precocious puberty among pediatric patients with HS. Screening may include assessment of height and weight, body mass index, blood pressure, fasting lipid levels, and glycated hemoglobin, as well as evaluation for signs and symptoms of PCOS or precocious puberty when clinically indicated [16,20]. A multidisciplinary healthcare team involving dermatologists, primary care physicians, mental health professionals, endocrinologists, and wound care specialists may facilitate earlier recognition and coordinated management of these comorbidities (Table 3) [19,20].

### 3.6. Racial/Ethnic Differences

A recurrent barrier to accessing high-quality dermatologic care for HS is the difference in disease presentation on various skin tones. A theme identified was the challenges experienced by Black pediatric patients with HS in the diagnosis and management of the disease, along with screening for comorbidities, compared to their White counterparts [20,21,22]. Recommendations to improve care include ensuring that families and healthcare providers receive adequate education on variations in disease presentation among different pediatric population groups (Table 3) [20,21,22].

### 3.7. HS Awareness

A recurrent theme highlighted across multiple studies was the prioritization of community and healthcare awareness of HS [3,17,18,20]. Without proper education or awareness, HS may go undiagnosed or falsely diagnosed. Among healthcare providers, especially primary care physicians and dermatologists, organizing multidisciplinary workshops focusing on the epidemiology and clinical presentation of HS to avoid delays in diagnosis and management for pediatric patients has been reported to be successful [18,19]. A possible modality of increasing awareness among diagnosed patients and their families is to incorporate HS support groups into care, as they act as a method to combat social isolation and allow patients to learn more about the diagnosis from people with similar experiences (Table 3) [17,18].

## 4. Discussion

This study aimed to better understand the patient experience in children and adolescents living with HS, including the social–emotional, physical, and mental health consequences associated with this dermatologic condition. Gaps in pediatric HS care, specifically in education, psychiatric resources, and medical intervention, were highlighted, along with the impact of such gaps on patient outcomes across different demographic groups. By investigating current shortcomings in HS care, the ultimate goal of this work is to improve the quality of life, mitigate the risk of comorbidities, and promote the social–emotional development of children growing up with HS.

HS is associated with prominent social concerns that can significantly impact the quality of life and social–emotional development of these patients. This study identified that children with HS experience social withdrawal related to peer stigma and feelings of shame or embarrassment. Even small social deviations such as the inability to shave one’s underarms in patients with axillary involvement of disease can promote these feelings of social isolation [3,15,18,19,20]. A 2024 multicenter cross-sectional study investigated the role of stigmatization in chronic pediatric skin disorders [6]. In this study, HS was identified as being associated with a high level of both experiences and perceived stigma. This was particularly interesting because, compared to other highly visible pediatric chronic skin conditions, the lesions of HS can typically be concealed by clothing. This suggests that the stigma and subsequent social consequences of stigma cannot be attributed to disease visibility. Social challenges become even more concerning as they result in avoidance of school, sports, and physical activity.

Moreover, emotional consequences are closely tied to social concerns. In the study of stigmatization of pediatric chronic skin conditions, there was a strong correlation between perceived stigma and mental health, particularly depression and anxiety [6]. Depression was the most frequently reported psychological comorbidity across the included studies. Children living in lower-socioeconomic-status (SES) environments may be particularly vulnerable to these social–emotional challenges. Lower SES has been associated with increased risk of HS, higher disease burden, and poorer quality of life, in part due to factors such as higher rates of smoking and obesity, limited access to healthy food, and reduced opportunities for physical activity [5]. These same neighborhood and household-level stressors can compound the psychosocial effects of HS in children, who may already struggle with stigma, embarrassment, and social withdrawal.

Lower-SES families also face greater barriers in accessing consistent dermatologic care, mental health services, and wound-care resources, which can lead to delayed treatment and worsening symptoms [24,25]. In turn, more severe or persistent disease may heighten feelings of shame, isolation, or avoidance of social and school activities [6]. Additionally, socioeconomic disadvantage is closely intertwined with racial and ethnic disparities seen in HS, and children from these backgrounds may encounter additional discrimination in both social and healthcare settings [25]. Together, these socioeconomic factors may amplify the emotional toll of the disease and contribute to a cycle in which limited resources, increased psychosocial stress, and greater disease severity reinforce one another.

Pediatric patients with HS also frequently experience physical health comorbidities, including metabolic and endocrine conditions, chronic pain, and other conditions. Obesity is the most consistently reported metabolic comorbidity, affecting about 37% of pediatric patients with HS, which is substantially higher than that of the general population. The relationship between HS and obesity is bidirectional, with obesity both contributing to and resulting from chronic inflammation in HS [26]. Endocrine disorders are notable, with type 2 diabetes affecting 4% of pediatric patients with HS [26]. PCOS and precocious puberty are also observed at higher rates than in the general population as well, with PCOS affecting up to 7% of pediatric patients with HS [27]. Hirsutism is present in about 14% [26].

Furthermore, one of the most impactful symptoms on quality of life is the chronic pain often experienced by patients with HS, often leading to increased emergency department utilization [27]. HS therefore exemplifies a biopsychosocial condition in which physical symptoms, psychological distress, and social functioning are deeply interconnected. Chronic pain and inflammatory comorbidities can intensify emotional symptoms such as anxiety and depression, while these mental health challenges in turn heighten pain perception and reduce coping capacity [28]. This reciprocal cycle often contributes to greater social withdrawal, decreased participation in school and activities, and further deterioration in quality of life. For pediatric patients, who are still developing socially and emotionally, these interactions are particularly impactful [27,28]. As such, comprehensive assessment that integrates dermatologic, psychological, and social factors is essential to fully address the multidimensional burden of HS and to support healthy developmental trajectories.

## 5. Clinical Implications

Given the substantial psychosocial and physical burden of HS in youth, these findings support the need for early and proactive screening for depression, anxiety, substance use, chronic pain, and metabolic abnormalities in pediatric patients. The high prevalence of comorbid obesity, endocrine disorders, and mental health concerns underscores the importance of a multidisciplinary approach that includes dermatology, primary care, endocrinology, pain management, and mental health professionals. Improved provider education is also critical, particularly regarding early and atypical presentations of HS and its appearance across diverse skin tones, as delayed or missed diagnoses disproportionately affect marginalized populations. Additionally, patient and family education, psychosocial support resources, and consistent follow-up can improve coping, reduce stigma, and enhance overall quality of life [3,28]. Integrating these strategies into routine pediatric HS care may mitigate long-term morbidity and better support healthy social–emotional development.

## 6. Limitations

This scoping review has several important limitations. First, the available literature on pediatric HS remains limited, with most studies drawing from small cohorts, case reports, or retrospective databases. As a result, the evidence may not fully capture the breadth of social, emotional, and physical consequences experienced by youth with HS. Second, the included studies demonstrated substantial heterogeneity in design, population characteristics, diagnostic criteria, and outcome measures, which limit the ability to compare findings across studies and may contribute to variability in reported associations. Third, the restriction to U.S.-based evidence substantially limited the number of eligible studies and reduces the international generalizability of the findings. The clinical and psychosocial consequences identified are not presumed to be unique to U.S. patients; rather, the U.S. focus reflects an effort to examine these outcomes within a shared healthcare and structural context. Future reviews incorporating international evidence are needed to determine which consequences are consistent across settings and which may be modified by healthcare systems, cultural factors, or social conditions. Additionally, many studies relied on administrative or electronic medical record datasets, where diagnostic coding inaccuracies and missing data may lead to underdiagnosis or misclassification of HS and its comorbidities. Furthermore, social–emotional outcomes were often measured inconsistently or not assessed using validated tools, reducing the precision of reported associations. Moreover, age definitions were not uniform across included studies, with some studies using broader age thresholds that extended into young adulthood. This heterogeneity may limit direct comparability across studies and should be considered when interpreting findings as specific to pediatric populations. Finally, racial, ethnic, and socioeconomic disparities were noted but not uniformly evaluated, limiting the ability to fully understand the role of structural factors in shaping outcomes for underserved pediatric populations. Despite these limitations, the findings highlight important patterns and underscore the urgent need for more comprehensive, prospective, pediatric-focused HS research.

## 7. Conclusions

HS imposes substantial psychosocial and physical burdens on children and adolescents, affecting their emotional well-being, social functioning, and overall social–emotional development. The interplay of stigma, chronic pain, and comorbid physical conditions underscores the importance of early diagnosis, as delayed recognition contributes to worsening symptoms and preventable psychosocial harm. Despite the significant impact of HS in youth, current clinical approaches are largely adapted from adult populations and do not adequately address the unique needs of pediatric patients. These findings underscore the urgent need for pediatric-specific guidelines that incorporate psychosocial assessment, early mental health support, and coordinated multidisciplinary care. Establishing such frameworks can improve clinical outcomes, reduce long-term morbidity, and support healthier social–emotional development for young patients growing up with HS.

## Figures and Tables

**Table 1:** Study Characteristics.

Article	Primary Author/Year	Study Design	Pediatric HS Sample Size	Study Population	Location in the US	Age Range	Study Purpose	Social Concerns Related to HS	Emotional/Physical Concerns Related to HS	Major Health Outcomes Resulting from HS
**1**	Cohn et al., 2024 [15]	Retrospective Cohort Study	*n* = 997	Pediatric patients with newly diagnosed HS attending 1 of 40 healthcare organizations across the US	New Hyde Park, NY	12–17 years old	To investigate the incidence and risk factors for anxiety in adolescents with HS	Social withdrawal from peers related to feelings of shame and embarrassment	Anxiety related to feelings of uncleanliness Impaired emotional development Pain and potential disfigurement	Increased risk of anxiety disorders Functional impairment related to psychosocial distress that may persist into adulthood
**2**	Cotton et al., 2023 [3]	Narrative Review	*n* = 5907	Pediatric patients with HS included in nine previously published studies	Washington, DC	≤18 years old	To summarize knowledge to guide clinicians in recognizing and treating pediatric HS	Limitation in patient ability to participate in sports Decreased ability to shave, thereby not complying with social standards Judgment from peers	Depression, anxiety, feelings of frustration Emotional distress in families caring for children with HS Physical pain from abscesses	Comorbidities including obesity, metabolic syndrome, PCOS, psychiatric disorders, Down syndrome, IBD, other skin conditions Overall reduced quality of life and mental wellbeing
**3**	Ghanshani et al., 2025 [16]	Narrative Review	N/A	Pediatric patients with HS	Los Angeles, CA	≤18 years old	To review the medical and surgical management of HS in pediatric patients	N/A	Increased anxiety and depression Some treatment options can be painful and will be variably tolerated by pediatric patients	Association with obesity, dyslipidemia, hypertension, PCOS, and diabetes Increased rates of other inflammatory conditions like IBD and spondylarthritis
**4**	Kirby et al., 2021 [17]	Editorial	N/A	Pediatric patients with HS	Hershey, PA	≤18 years old	To discuss the commonality, disparity, severity, and management of HS in children	Disease burden impacts family members, thereby affecting family relationships	Depression rates double that of the general population	Association with obesity and other comorbidities Hormonal imbalances resulting in conditions such as PCOS and precocious puberty Racial disparities in access to care
**5**	Lee et al., 2022 [18]	Narrative Review	N/A	Pediatric patients with HS	San Diego, CA	≤18 years old	To address the diagnosis, epidemiology, associated comorbidities, and management of HS in the pediatric population	Social isolation Stigmatization Sociodemographic-related barriers to timely care, particularly seen in minority communities	Increased rate of depression Poor self-esteem	Chronic pain Association with comorbidities including obesity, diabetes, and IBD Poor outcomes due to barriers to care and treatment delays
**6**	Power et al., 2024 [19]	Retrospective Cohort Study	*n* = 9	Patients with axillary Hurley stage 3 HS who were seen in a pediatric surgery clinic	New Orleans, LA	≤21 years old	To determine if a staged surgical approach for treatment of axillary Hurley Stage 3 HS can eradicate disease and improve quality of life	Remaining homebound Avoidance of career opportunities Not attending in-person school	Suicidal ideation, depression, and hopelessness Overall diminished mental health	Diminished mental health Decreased range of motion Decreased perceived quality of life
**7**	Seivright et al., 2021 [20]	Retrospective Cohort Study	*n* = 73	Pediatric HS patients from the University of California Los Angeles health system	Los Angeles, CA	<25 years old	To explore physical and psychosocial comorbidities in pediatric HS patients	Decreased social functioning and quality of life affecting everyday life	Psychiatric problems including anxiety, depression, substance use disorder, suicidal ideation Metabolic and endocrine comorbidities (i.e., obesity and PCOS) Pulmonary diseases (i.e., asthma)	Prevalent comorbidities including metabolic, psychiatric, and other dermatologic concerns such as acne vulgaris, acanthosis nigricans, atopic dermatitis
**8**	Singal et al., 2024 [21]	Cohort Study	*n* = 1290	Pediatric patients with HS	National	≤21 years old	To explore the racial discrepancies in demographics and comorbidities of pediatric HS patients	Black children with HS had lower family income, increased use of Medicaid, decreased utilization of outpatient dermatology care, lower hospital charges, and shorter hospital stays	Increased rate of depression and anxiety Painful cutaneous abscess, pilonidal cyst Other comorbidities including asthma and obesity	Evidence of disparities in care for black children with HS resulting in fewer comorbid diagnoses documented, more cutaneous abscesses and asthma suggesting potential underdiagnosis and undertreatment
**9**	Wright et al., 2020 [22]	Retrospective Cohort study	*n* = 3042	Pediatric patients with HS	National	10–17 years old	To explore the risk of developing depression among patients with HS compared to those without HS	Increased rates of smoking, alcohol use disorder, and substance use disorder	Increased rate of depression Increased rate of obesity	Higher risk of developing depression independent of risk factors such as obesity, smoking, alcohol/substance use disorder For every 100 HS patients without pre-existing depression, 3 new cases of depression are diagnosed each year
**10**	Wright et al., 2022 [23]	Cross-Sectional Study	*n* = 1162	Pediatric patients with HS seen across 4 census regions in hospitals or other healthcare settings	National	10–17 years old	To explore the prevalence of depression in adults, adolescents, and children with HS compared to controls	Alcohol and substance use disorder Increased smoking	Increased rate of depression Increased rate of obesity	Increased prevalence of depression among HS patients compared to controls Patients with HS are commonly affected by depression and anxiety

**Table 2:** Association Between HS and Social–Emotional Concerns.

Article	Primary Author/Year	Statistical Analysis Carried Out	Significance of Association	Database Used (If Not Database, How Was the Data Collected)
**1**	Cohn et al., 2024 [15]	Cox proportional hazard regression used to compare crude risk of anxiety disorder between groups and assess the independent association with HS while controlling for confounders	Significant (female sex, black race, history of depression). Non-significant (age, insurance type, BMI category, frequency of outpatient/ED visits).	IBM Explorys Life Sciences Database
**2**	Cotton et al., 2023 [3]	N/A	N/A	N/A
**3**	Ghanshani et al., 2025 [16]	N/A	N/A	N/A
**4**	Kirby et al., 2021 [17]	N/A	N/A	N/A
**5**	Lee et al., 2022 [18]	N/A	N/A	N/A
**6**	Power et al., 2024 [19]	Student’s *t*-test or Fischer’s exact test used to compare patient characteristics between surgical and medical management groups	Non-Significant (gender, age, BMI, insurance type, age at HS diagnosis, duration of disease prior to surgical encounter, HS ED encounters, incision and drainage procedures)	Single-center pediatric clinical database, Children’s Hospital New Orleans
**7**	Seivright et al., 2021 [20]	Chi-square tests used to evaluate comorbidity associations with gender, race, and pre- vs. post-teen onset *t*-test to evaluate comorbidity associations with BMI Chi-square GOF tests were used to compare HS comorbidities vs. general US pediatric population	Significant (acne vulgaris, acanthosis nigricans, seborrheic dermatitis, pilonidal cyst, psoriasis, Down syndrome, irregular periods, PCOS, hirsutism, precocious puberty, elevated serum testosterone, obesity, pre-diabetes, diabetes, anxiety, depression, suicidal ideation, substance use disorder, and asthma and other reactive airway disorders). Non-significant (atopic dermatitis, hypercholesterolemia, hypertension, ADHD, autism, bipolar disorder)	Electronic health record from University of California Los Angeles health system
**8**	Singal et al., 2024 [21]	SPSS was used to collect and analyze demographic differences and comorbidity prevalence among pediatric HS patients of Black vs. other races	Significant (obesity, asthma, anxiety, and cutaneous abscess). Non-significant (anemia, depression, hypertension, sepsis, pilonidal cyst, and PCOS).	2016 Kids’ Inpatient Database (KID)
**9**	Wright et al., 2020 [22]	Cox proportional hazards regression model with adjusted hazard ratio to compare risk of depression among patients with HS vs. controls; a robust variance estimator was used to account for clustering Cox proportional hazards regression model to evaluate demographic and clinical factors associated with depression within the pediatric and adult HS cohorts 2 sensitivity analyses: (1) Propensity score matching using unadjusted Cox proportional hazards regression model with a robust variance estimator; (2) complete case analysis excluding patients with any missing data	Significant (ever smoker vs. never smoker and obese). Non-significant (substance use disorder, underweight, and overweight).	IBM Explorys Life Sciences Database
**10**	Wright et al., 2022 [23]	Conditional logistic regression model, adjusting for age, sex, race, number of healthcare encounters within the study period, smoking status (ever, never), alcohol use disorder, substance use disorder, body mass index, and Charlson comorbidity index score Unadjusted and demographic-adjusted logistic regression models used to assess the influence of covariates on the relationship between HS and depression	Significant (depression among adult HS patients) and Non-significant (depression among pediatric HS patients)	IBM Explorys Life Sciences Database

**Table 3:** Recommendations to inform future research, public health efforts, or regulatory guidance on pediatric HS.

Article	Primary Author/Year	Future Recommendations to Inform Future Research, Public Health Efforts, or Regulatory Guidance on Pediatric HS	Major Recommendations Across Studies
**1**	Cohn et al., 2024 [15]	Screen adolescents with HS for anxiety disorders and implement early interventions	Increase screening practices for recognized HS-related comorbidities including: Depression Anxiety Decreased quality-of-life Metabolic conditions (obesity, PCOS, precocious puberty, metabolic syndrome) Prioritize early recognition and management of HS in pediatric patients Pursue multimodal treatment regimens Recognize potential discrepancies in care affecting pediatric HS patients, particularly Black individuals Improve HS awareness among providers, family members, and the broader community
**2**	Cotton et al., 2023 [3]	Further research specific to pediatric populations Integration of quality-of-life measures into clinical practice Improved early recognition and management of HS in pediatric patients	
**3**	Ghanshani et al., 2025 [16]	Beginning multimodal treatment early in pediatric patients with HS and appropriately screening for known comorbidities	
**4**	Kirby et al., 2021 [17]	Further controlled HS studies not limited to retrospective medical record review Future studies in HS should ensure to include quality of life and psychological screening Studies in pediatric patients should include long-term follow-up Better HS awareness in families and communities	
**5**	Lee et al., 2022 [18]	Establish HS support groups to combat social isolation Increase support in transitioning older adolescents into adult care Increase HS education among pediatric providers	
**6**	Power et al., 2024 [19]	Due to the limitations in medical management of HS, a staged surgical approach should be considered This study focused on axillary lesions only. Future work should consider other locations of HS Establish interdisciplinary care for HS patients with dermatology, wound care, and psychology	
**7**	Seivright et al., 2021 [20]	Standardized screening of mental health and other comorbidities in pediatric HS patients regardless of gender, racial background, or HS severity Metabolic screening regardless of preexisting obesity (height and weight, blood pressure, fasting lipid panel, glycated hemoglobin, precocious puberty, PCOS) Research exploring the driving mechanisms of atopic disease co-occurrence in patients with HS is necessary Research looking at the unique challenges that pediatric HS patients face– including impacts on quality of life Need prospective, large-scale studies of understudied and underreported HS comorbidities	
**8**	Singal et al., 2024 [21]	The study findings suggest potential discrepancies in care of Black pediatric HS patients that require further research Retrospective design Recommend investigating discrepancies in pharmacologic treatments among Black pediatric HS patients	
**9**	Wright et al., 2020 [22]	Periodic depression screenings among patients with HS, especially women, smokers, and those with substance use disorder	
**10**	Wright et al., 2022 [23]	Periodic depression screenings for patients with HS Providers should be attentive to signs and symptoms of depression in all patients with HS Future studies should investigate the impact of HS severity on depression	

## Data Availability

No new data were created or analyzed in this study. Data sharing is not applicable to this article.

## Supplementary Materials

The following supporting information can be downloaded at: https://www.mdpi.com/article/10.3390/pediatric18040103/s1, Table S1: Preferred Reporting Items for Systematic reviews and Meta-Analyses extension for Scoping Reviews (PRISMA-ScR) Checklist [11]; File S1: Search Strategy.
