# Supplementary material for: Exploring the Social, Emotional, and Physical Consequences of Hidradenitis Suppurativa in Pediatric Patients: A Scoping Review
Source: Pediatr Rep. 2026 Aug 4;18(4):103. doi: 10.3390/pediatric18040103 (PMC13516647; doi:10.3390/pediatric18040103)
Supplement: Supplementary file 1 [file pediatrrep-18-00103-s001.zip › pediatrrep-4416684-File S1-Search Strategy.pdf]

Database: PubMed.gov (Includes MEDLINE)

("hidradenitis suppurativa"[MeSH Terms] OR "acne inversa"[Title/Abstract] OR "Apocrinitis"[Title/Abstract] OR "Hidradenitis"[Title/Abstract] OR "hidradenitis suppurativa"[Title/Abstract] OR "suppurative hidradenitis"[Title/Abstract] OR "velpeau disease"[Title/Abstract] OR "velpeau s disease"[Title/Abstract] OR "verneuil disease"[Title/Abstract] OR "verneuil s disease"[Title/Abstract]) AND ("adolescent"[MeSH Terms] OR "child"[MeSH Terms] OR "pediatrics"[MeSH Terms] OR "young adult"[MeSH Terms] OR "adolescen*"[Title/Abstract] OR "child*"[Title/Abstract] OR "Infant"[Title/Abstract] OR "Juvenile"[Title/Abstract] OR "paediatric*"[Title/Abstract] OR "pediatric*"[Title/Abstract] OR "teen*"[Title/Abstract] OR "Young"[Title/Abstract] OR "Youth"[Title/Abstract]) AND ("anxiety"[MeSH Terms] OR "attention deficit disorder with hyperactivity"[MeSH Terms] OR "bullying"[MeSH Terms] OR "depression"[MeSH Terms] OR "depressive disorder"[MeSH Terms] OR "disruptive, impulse control, and conduct disorders"[MeSH Terms] OR "mental disorders"[MeSH Terms] OR "mental health"[MeSH Terms] OR "mood disorders"[MeSH Terms] OR "neurocognitive disorders"[MeSH Terms] OR "neurodevelopmental disorders"[MeSH Terms] OR "obsessive compulsive disorder"[MeSH Terms] OR "personality disorders"[MeSH Terms] OR "psychotic disorders"[MeSH Terms] OR "quality of life"[MeSH Terms] OR "social behavior"[MeSH Terms] OR "social stigma"[MeSH Terms] OR "stereotyping"[MeSH Terms] OR "substance related disorders"[MeSH Terms] OR "Antidepressants"[Title/Abstract] OR "Antipsychotics"[Title/Abstract] OR "anxi*"[Title/Abstract] OR "attention deficit disorder with hyperactivity"[Title/Abstract] OR "ADHD"[Title/Abstract] OR "Bipolar"[Title/Abstract] OR "bully*"[Title/Abstract] OR "depress*"[Title/Abstract] OR "eating disorder*"[Title/Abstract] OR "emot*"[Title/Abstract] OR "mental disorder*"[Title/Abstract] OR "mental health"[Title/Abstract] OR "mood disorder*"[Title/Abstract] OR "quality of life"[Title/Abstract] OR "OCD"[Title/Abstract] OR "obsessive compulsive disorder"[Title/Abstract] OR "Panic"[Title/Abstract] OR "personality disorder"[Title/Abstract] OR "phobia*"[Title/Abstract] OR "post traumatic stress disorder"[Title/Abstract] OR "PTSD"[Title/Abstract] OR "psych*"[Title/Abstract] OR "schizophreni*"[Title/Abstract] OR "sexual dysfunction"[Title/Abstract] OR "sleep disorders"[Title/Abstract] OR "social*"[Title/Abstract] OR "stereotyp*"[Title/Abstract] OR "stigma*"[Title/Abstract] OR "suicid*"[Title/Abstract])

Database: Embase.com (Elsevier)

('suppurative hidradenitis'/exp OR 'acne inversa':ti,ab OR Apocrinitis:ti,ab OR Hidradenitis:ti,ab OR 'hidradenitis suppurativa':ti,ab OR 'suppurative hidradenitis':ti,ab OR 'velpeau disease':ti,ab OR 'velpeau s disease':ti,ab OR 'verneuil disease':ti,ab OR 'verneuil

s disease':ti,ab) AND (adolescent/exp OR child/exp OR pediatrics/exp OR 'young adult'/exp OR adolescen*:ti,ab OR child*:ti,ab OR Infant:ti,ab OR Juvenile:ti,ab OR paediatric*:ti,ab OR pediatric*:ti,ab OR teen*:ti,ab OR Young:ti,ab OR Youth:ti,ab) AND (anxiety/exp OR 'attention deficit disorder with hyperactivity'/exp OR bullying/exp OR depression/exp OR 'disorders of higher cerebral function'/exp OR 'disruptive disorder'/exp OR 'drug dependence'/exp OR 'impulse control disorder'/exp OR 'mental disease'/exp OR 'mental health'/exp OR 'mood disorder'/exp OR 'obsessive compulsive disorder'/exp OR 'personality disorder'/exp OR 'psychosis'/exp OR 'quality of life'/exp OR 'social behavior'/exp OR stereotyping/exp OR Antidepressants:ti,ab OR Antipsychotics:ti,ab OR anxi*:ti,ab OR 'attention deficit disorder with hyperactivity':ti,ab OR ADHD:ti,ab OR Bipolar:ti,ab OR bully*:ti,ab OR depress*:ti,ab OR 'eating disorder*':ti,ab OR emot*:ti,ab OR 'mental disorder*':ti,ab OR 'mental health':ti,ab OR 'mood disorder*':ti,ab OR 'quality of life':ti,ab OR OCD:ti,ab OR 'obsessive compulsive disorder':ti,ab OR Panic:ti,ab OR 'personality disorder':ti,ab OR phobia*:ti,ab OR 'post traumatic stress disorder':ti,ab OR PTSD:ti,ab OR psych*:ti,ab OR schizophreni*:ti,ab OR 'sexual dysfunction':ti,ab OR 'sleep disorders':ti,ab OR social*:ti,ab OR stereotyp*:ti,ab OR stigma*:ti,ab OR suicid*:ti,ab)

Database: Cochrane Library

([mh "hidradenitis suppurativa"] OR "acne inversa":ti,ab OR Apocrinitis:ti,ab OR Hidradenitis:ti,ab OR "hidradenitis suppurativa":ti,ab OR "suppurative hidradenitis":ti,ab OR "velpeau disease":ti,ab OR "velpeau s disease":ti,ab OR "verneuil disease":ti,ab OR "verneuil s disease":ti,ab) AND ([mh adolescent] OR [mh child] OR [mh pediatrics] OR [mh "young adult"] OR adolescen*:ti,ab OR child*:ti,ab OR Infant:ti,ab OR Juvenile:ti,ab OR paediatric*:ti,ab OR pediatric*:ti,ab OR teen*:ti,ab OR Young:ti,ab OR Youth:ti,ab) AND ([mh anxiety] OR [mh "attention deficit disorder with hyperactivity"] OR [mh bullying] OR [mh depression] OR [mh "depressive disorder"] OR [mh "disruptive, impulse control, and conduct disorders"] OR [mh "mental disorders"] OR [mh "mental health"] OR [mh "mood disorders"] OR [mh "neurocognitive disorders"] OR [mh "neurodevelopmental disorders"] OR [mh "obsessive compulsive disorder"] OR [mh "personality disorders"] OR [mh "psychotic disorders"] OR [mh "quality of life"] OR [mh "social behavior"] OR [mh "social stigma"] OR [mh stereotyping] OR [mh "substance related disorders"] OR Antidepressants:ti,ab OR Antipsychotics:ti,ab OR anxi*:ti,ab OR "attention deficit disorder with hyperactivity":ti,ab OR ADHD:ti,ab OR Bipolar:ti,ab OR bully*:ti,ab OR depress*:ti,ab OR ("eating" NEXT disorder*):ti,ab OR emot*:ti,ab OR ("mental" NEXT disorder*):ti,ab OR "mental health":ti,ab OR ("mood" NEXT disorder*):ti,ab OR "quality of life":ti,ab OR OCD:ti,ab OR "obsessive compulsive disorder":ti,ab OR Panic:ti,ab OR "personality

disorder":ti,ab OR phobia*:ti,ab OR "post traumatic stress disorder":ti,ab OR PTSD:ti,ab OR psych*:ti,ab OR schizophreni*:ti,ab OR "sexual dysfunction":ti,ab OR "sleep disorders":ti,ab OR social*:ti,ab OR stereotyp*:ti,ab OR stigma*:ti,ab OR suicid*:ti,ab)

Database: Scopus

(INDEXTERMS("hidradenitis suppurativa") OR TITLE-ABS("acne inversa") OR TITLE-ABS(Apocrinitis) OR TITLE-ABS(Hidradenitis) OR TITLE-ABS("hidradenitis suppurativa") OR TITLE-ABS("suppurative hidradenitis") OR TITLE-ABS("velpeau disease") OR TITLE-ABS("velpeau s disease") OR TITLE-ABS("verneuil disease") OR TITLE-ABS("verneuil s disease")) AND (INDEXTERMS(adolescent) OR INDEXTERMS(child) OR INDEXTERMS(pediatrics) OR INDEXTERMS("young adult") OR TITLE-ABS(adolescen*) OR TITLE-ABS(child*) OR TITLE-ABS(Infant) OR TITLE-ABS(Juvenile) OR TITLE-ABS(paediatric*) OR TITLE-ABS(pediatric*) OR TITLE-ABS(teen*) OR TITLE-ABS(Young) OR TITLE-ABS(Youth)) AND (INDEXTERMS(anxiety) OR INDEXTERMS("attention deficit disorder with hyperactivity") OR INDEXTERMS(bullying) OR INDEXTERMS(depression) OR INDEXTERMS("depressive disorder") OR INDEXTERMS("disruptive, impulse control, and conduct disorders") OR INDEXTERMS("mental disorders") OR INDEXTERMS("mental health") OR INDEXTERMS("mood disorders") OR INDEXTERMS("neurocognitive disorders") OR INDEXTERMS("neurodevelopmental disorders") OR INDEXTERMS("obsessive compulsive disorder") OR INDEXTERMS("personality disorders") OR INDEXTERMS("psychotic disorders") OR INDEXTERMS("quality of life") OR INDEXTERMS("social behavior") OR INDEXTERMS("social stigma") OR INDEXTERMS(stereotyping) OR INDEXTERMS("substance related disorders") OR TITLE-ABS(Antidepressants) OR TITLE-ABS(Antipsychotics) OR TITLE-ABS(anxi*) OR TITLE-ABS("attention deficit disorder with hyperactivity") OR TITLE-ABS(ADHD) OR TITLE-ABS(Bipolar) OR TITLE-ABS(bully*) OR TITLE-ABS(depress*) OR TITLE-ABS("eating disorder*") OR TITLE-ABS(emot*) OR TITLE-ABS("mental disorder*") OR TITLE-ABS("mental health") OR TITLE-ABS("mood disorder*") OR TITLE-ABS("quality of life") OR TITLE-ABS(OCD) OR TITLE-ABS("obsessive compulsive disorder") OR TITLE-ABS(Panic) OR TITLE-ABS("personality disorder") OR TITLE-ABS(phobia*) OR TITLE-ABS("post traumatic stress disorder") OR TITLE-ABS(PTSD) OR TITLE-ABS(psych*) OR TITLE-ABS(schizophreni*) OR TITLE-ABS("sexual dysfunction") OR TITLE-ABS("sleep disorders") OR TITLE-ABS(social*) OR TITLE-ABS(stereotyp*) OR TITLE-ABS(stigma*) OR TITLE-ABS(suicid*))

Database: Web of Science

(ALL="hidradenitis suppurativa" OR (TI="acne inversa" OR AB="acne inversa") OR (TI=Apocrinitis OR AB=Apocrinitis) OR (TI=Hidradenitis OR AB=Hidradenitis) OR (TI="hidradenitis suppurativa" OR AB="hidradenitis suppurativa") OR (TI="suppurative hidradenitis" OR AB="suppurative hidradenitis") OR (TI="velpeau disease" OR AB="velpeau disease") OR (TI="velpeau s disease" OR AB="velpeau s disease") OR (TI="verneuil disease" OR AB="verneuil disease") OR (TI="verneuil s disease" OR AB="verneuil s disease")) AND (ALL=adolescent OR ALL=child OR ALL=pediatrics OR ALL="young adult" OR (TI=adolescen* OR AB=adolescen*) OR (TI=child* OR AB=child*) OR (TI=Infant OR AB=Infant) OR (TI=Juvenile OR AB=Juvenile) OR (TI=paediatric* OR AB=paediatric*) OR (TI=pediatric* OR AB=pediatric*) OR (TI=teen* OR AB=teen*) OR (TI=Young OR AB=Young) OR (TI=Youth OR AB=Youth)) AND (ALL=anxiety OR ALL="attention deficit disorder with hyperactivity" OR ALL=bullying OR ALL=depression OR ALL="depressive disorder" OR ALL="disruptive, impulse control, and conduct disorders" OR ALL="mental disorders" OR ALL="mental health" OR ALL="mood disorders" OR ALL="neurocognitive disorders" OR ALL="neurodevelopmental disorders" OR ALL="obsessive compulsive disorder" OR ALL="personality disorders" OR ALL="psychotic disorders" OR ALL="quality of life" OR ALL="social behavior" OR ALL="social stigma" OR ALL=stereotyping OR ALL="substance related disorders" OR (TI=Antidepressants OR AB=Antidepressants) OR (TI=Antipsychotics OR AB=Antipsychotics) OR (TI=anxi* OR AB=anxi*) OR (TI="attention deficit disorder with hyperactivity" OR AB="attention deficit disorder with hyperactivity") OR (TI=ADHD OR AB=ADHD) OR (TI=Bipolar OR AB=Bipolar) OR (TI=bully* OR AB=bully*) OR (TI=depress* OR AB=depress*) OR (TI="eating disorder*" OR AB="eating disorder*") OR (TI=emot* OR AB=emot*) OR (TI="mental disorder*" OR AB="mental disorder*") OR (TI="mental health" OR AB="mental health") OR (TI="mood disorder*" OR AB="mood disorder*") OR (TI="quality of life" OR AB="quality of life") OR (TI=OCD OR AB=OCD) OR (TI="obsessive compulsive disorder" OR AB="obsessive compulsive disorder") OR (TI=Panic OR AB=Panic) OR (TI="personality disorder" OR AB="personality disorder") OR (TI=phobia* OR AB=phobia*) OR (TI="post traumatic stress disorder" OR AB="post traumatic stress disorder") OR (TI=PTSD OR AB=PTSD) OR (TI=psych* OR AB=psych*) OR (TI=schizophreni* OR AB=schizophreni*) OR (TI="sexual dysfunction" OR AB="sexual dysfunction") OR (TI="sleep disorders" OR AB="sleep disorders") OR (TI=social* OR AB=social*) OR (TI=stereotyp* OR AB=stereotyp*) OR (TI=stigma* OR AB=stigma*) OR (TI=suicid* OR AB=suicid*))

Date limiter for all searches: 2015 - 2025
